# Tobacco Smoke Carcinogens Induce DNA Repair Machinery Function Loss: Protection by Carbon Nanotubes

**DOI:** 10.31557/APJCP.2020.21.10.3099

**Published:** 2020-10

**Authors:** Anukriti Dhasmana, Anupam Dhasmana, Hobani Yahya H, Abdullah Farasani, Mahmoud Habibullah, Freah L Alshammary, Saif Khan, Shafiul Haque, Mohtashim Lohani

**Affiliations:** 1 *Himalayan School of Biosciences, Swami Rama Himalayan University, Dehradun (Uttarakhand), India. *; 2 *University of Texas Rio Grande Valley, McAllen, United States of America. *; 3 *Dean, Faculty of Applied Medical Sciences, Jazan University, Jazan, Saudi Arabia. *; 4 *Department of Medical Laboratory Technology, Faculty of Applied Medical Sciences, Jazan University, Jazan, Saudi Arabia. *; 5 *Emergency Medical Services Department, Faculty of Applied Medical Sciences, Jazan University, Jazan, Saudi Arabia. *; 6 *Department of Preventative Dental Sciences, College of Dentistry, Hail University, Hail, Saudi Arabia. *; 7 *Research and Scientific Studies Unit, Faculty of Nursing and Allied Health Sciences, Jazan University, Jazan, Saudi Arabia. *; 8 *Medical Research centre, Faculty of Applied Medical Sciences, Jazan University, Jazan, KSA. *

**Keywords:** Cancer, DNA damage/repair, TSNs, NNK, NNAL, molecular docking, nanoparticles

## Abstract

**Purpose:**

DNA damage is a continuous process occurring within the cells caused by intrinsic and extrinsic factors, but it gets repaired regularly. If the DNA repair process is faulty, the incidences of damages/mutations can accumulate in cells resulting in cell transformation. It is hypothesized that the negative variations in DNA repair pathways in even at one point viz. genetic, translational or posttranslational stage may fairly be crucial for the beginning and development of carcinogenesis. Therefore, we investigated the potential of tobacco specific nitrosamines (TSNs) related carcinogens to interact with the enzymes involved in DNA repair mechanisms in the current study.

**Methods:**

The derivatives of cigarettes’ smoke like NNK and NNAL are very well known and recognized carcinogens. Therefore, almost 120 enzymes playing crucial role in the DNA repair process have been analysed for their reactivity with NNK and NNAL.

**Results:**

The molecular docking study helped to screen out, 07 possible DNA repair enzyme targets for NNK, and 12for NNAL. Present study revealed the loss of activity of DNA repair enzymes in the presence of NNK and NNAL, and this accumulation may induce the tendency of DNA damage which can lead the transformation of exposed normal cells in to cancerous cells. This study also demonstrated the protective potential of nanoparticles like SWCNTs/MWCNTs against TSN’s induced toxicity; here SWCNT against NNK (-17.16 Kcal/Mol) and MWCNT against NNK -17.01 Kcal/Mol were showing maximum binding affinities than the known biomolecular target of NNK 1UGH (Uracil-DNA glycosylase,-7.82Kcal/Mol).

**Conclusion:**

CNTs can be applied as chemo-preventive agents against environmental and tobacco induced carcinogens owing to their scavenging potential and warrants for in vivo and in vitro experimental validation of the results obtained from the present study.

## Introduction

DNA damage and injury are continuous events that occurs in cells and are repaired also incessantly by DNA repair mechanisms. The accumulation of damages/mutations are necessary in a cell to undergo transformation. This is possible if the mutations escape the repair mechanisms of the cell. Various studies have shown that DNA damage because of external factors as well as internal metabolic processes occur at a rate of up to one million molecular damages per cell per day. The damaged critical genes (e.g. tumor suppressor genes/ proto-oncogenes) can obstruct the ability of cell to carry out its function and substantially escalate the probability of cell transformation. This appears feasible if the DNA repair process/s is/are hindered. Therefore, it was put forward that for the origination and advancement of cancer, impairment of the DNA repair process at any point like genetic, translational or post translational level, becomes fairly imperious (Gina etal., 2009). Various chemicals including cigarette smoke carcinogens [i.e., tobacco specific nitrosamines (TSNs)] like NNK (4-(Methylnitrosamino)-1-(3-pyridyl)-1-butanone) and NNAL (4-(methylnitrosamino)-1-(3-pyridyl)-1-butan-1-ol) make non repairable DNA lesions (Huang et al., 2011), and were therefore analysed for their actions on important enzymes in DNA repair machinery (Saito et al., 2012). NNK, a derivative of tobacco alkaloids ,is one of the established strong carcinogen in humans (Hecht and Hoffmann, 1989).

The present study involves simulation procedures to explore the binding efficiency of NNK and NNAL with the enzymes important in DNA repair processes. There are almost 120 enzymes engaged in DNA repair mechanisms (Dhasmana et al., 2015). Hence, in this study we planned to explore if chemicals like NNK and its metabolite NNAL, despite straight damaging the DNA, are affecting the DNA repair process also by interacting with these enzymes?

Lately, many nanoparticles have shown their scavenging effects for the reclamation of soil contaminated with heavy PAHs(polycyclic aromatic hydrocarbons) (Karnchanasest and Santisukkasaem, 2007). The rummaging abilities of nanoparticles towards PAHs and other toxicants can be attributed to surface chemistry, large and complimentary surface area and other inherent assets. Titanate Nano Tubes (TNT) and Titanate Nano Sheets (TNS) were employed as additives to get rid of detrimental chemicals from cigarette smoke (Qixin et al., 2011), and were found to successfully remove tar, hydrogen cyanide, nicotine, ammonia, phenolic compounds and selected carbonyls. Single and multi-walled Carbon nanotubes (CNTs) are gaining lot of attention from the scientific community owing to their extraordinary physical and atomic properties. The void one-dimensional and one-atom-thick structure of SWCNTs and more than one-atom-thick structure of MWCNTs are particularly great for adsorption associated applications. CNTs are widely used nowadays for creating sensors of pollutant smokes. Also, CNTs have been used for eliminating dangerous pollutants from gas streams because of their adsorption potentials (Agnihotri et al., 2005).

In view of the above mentioned characteristics of carbon nanotubes, the current study has been deliberated to investigate if CNTs could be exploited to defend the biological system against the harmful effects of TSNs exposure. The current study was performed using various bioinformatic tools for in silico experimentations to achieve a deep insight of the mechanism of protection.

## Materials and Methods

**Figure 1:**
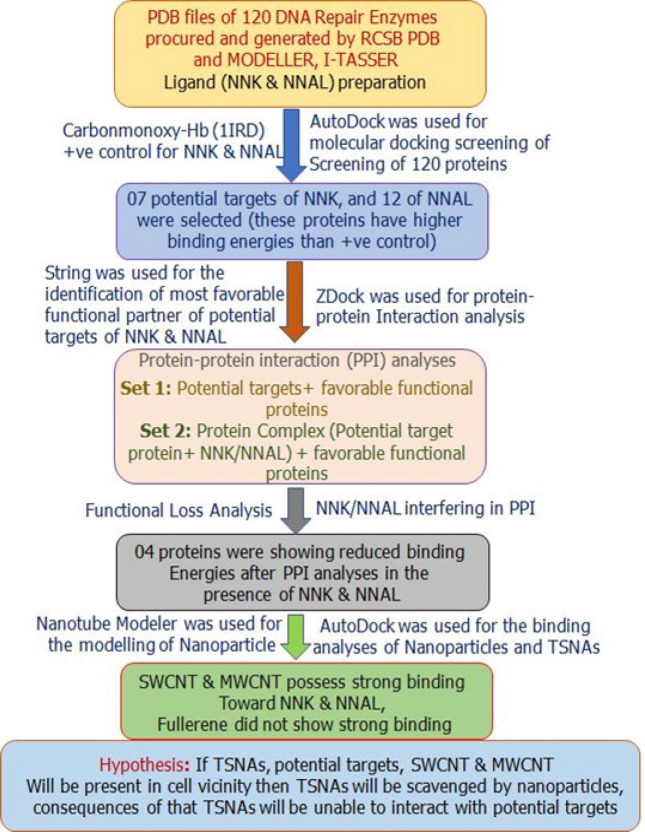
Schematic Methodology of the Analyses

Schematic flowchart of methodology is discussed in the Figure 1.

*Ligand structures preparation*

**Figure 2:**
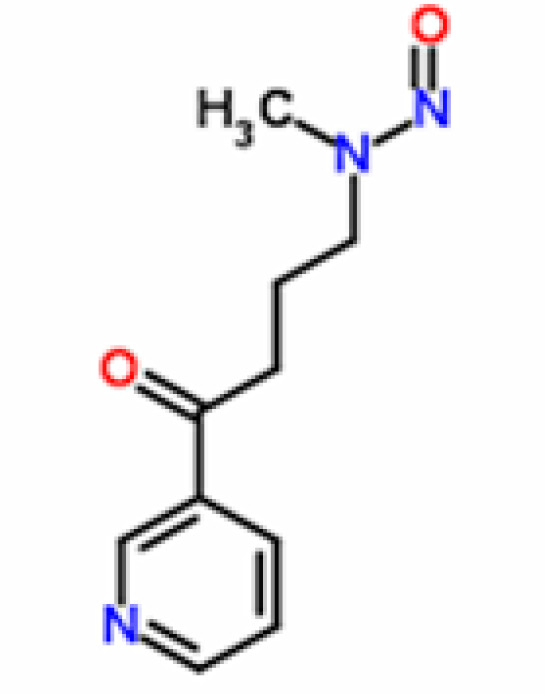
Chemical Structure of NNK [4-(Methylnitrosamino)-1-(3-pyridyl)-1-butanone] PubChem Compound ID- 47289, ChemSpider ID-43038

**Figure 3:**
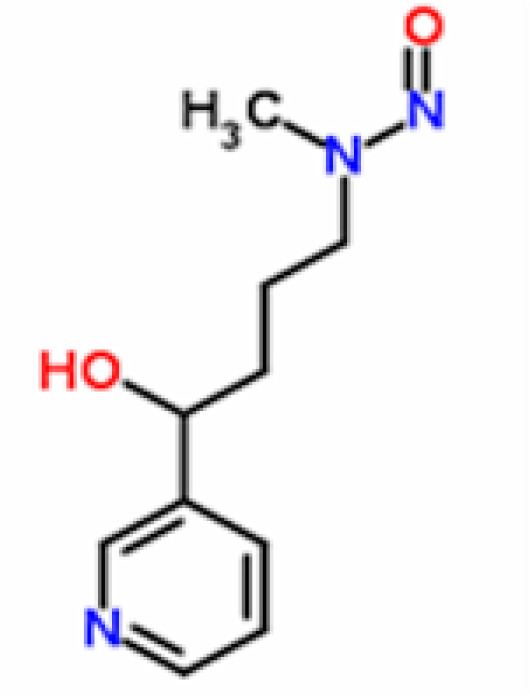
Chemical structure of NNAL [4-(methylnitrosamino)-1-(3-pyridyl)-1-butan-1-ol] PubChem Compound ID- 104856, ChemSpider ID- 94646

We downloaded the ligand files of NNK (4-(Methylnitrosamino)-1-(3-pyridyl)-1-butanone) and NNAL (4-(methylnitrosamino)-1-(3-pyridyl)-1-butan-1-ol) from ChemSpider Chemical Database in .mol format (Figures 2 and 3). These files were then converted to .pdb files with help of Discovery Studio Visualizer , as AutoDock 4.0 cannot read .mol files (Morris et al., 1996). Further, these ligands were submitted for energy minimization in Chimera Version 1.5.3 with Genetic Algorithm, 0.5 grid units optimized and steps 2000 (Pettersen et al., 2004).

*Preparation of protein structures*

The structures of all enzymes involved in DNA repair process, used in the current study, were acquired from Protein Data Bank RCSB. Those structures of enzymes, which were not found in there, were obtained from I-Tasser and Homology Modelling-Modeler 9.10 online server (Eswar et al., 2006; Roy et al., 2010). The modelled protein structures had already been mentioned in the study of Dhasmana et al., (2015). The published structures were amended to eliminate HETATM with help of Discovery Studio Visualizer. The energy was minimized, steric collision was removed with the sharpest descent steps 1000, sharpest descent size 0.02 Å, conjugated gradient steps 1000 and the conjugate gradient step size 0.02 Å for the conjugate gradient minimization with help of Chimera (Wang et al., 2004; 2006).

*Procurement of carbon based nanotube and fullerene*

**Figure 4:**
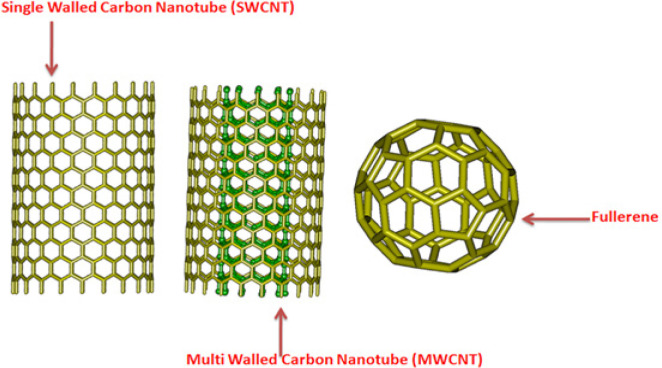
3D Structure of SWCNT, MWCNT and Fullerene

The designing and procurement of .pdb files of carbon-based nanoparticle (Figure 4) was done using Nanotube Modeler software program (Firdaus et al., 2017). The parameters of the designed single walled carbon nanotubes (SWCNTs) were bond length 1.421nm, tube length 2.5nm, whereas the parameters of multi walled carbon nanotubes (MWCNTs) were with bond length 1.421 nm, two wall layers and tube length 2.5 nm, while the parameter of fullerene was with diameter of 1nm.

*Molecular docking studies*

The docking evaluations were executed by using AutoDock 4.0 suite based on genetic algorithm (GA) (Morris et al., 2009; Rarey et al., 1996). The Cygwin interface was used with 10 times run of each docking, in Intel (R) i7-5500U, CPU 2.40 GHz and 16.0 GB of RAM of DELL machine. The molecular docking method was implemented followed by searching the best conformation of DNA repair enzymes, both CNTs, fullerene, NNK and NNAL complexes on the basis of molecular binding energy. All AutoDock 4.0 parameters were used for this study have been discussed in our previously published article (Dhasmana et al., 2015). For the characterization of the docked binding sites of Protein-Ligands complex, the LigSite online server was used (Hendlich et al., 1997) (Table 1). The most favourable functional partners of enzymes involved in DNA repair process were obtained with help of STRING 9.0. The known database (http://string-db.org/) for predicting protein-protein interactions was used for predicting the interaction of the query molecules. The ZDOCK protein-protein docking program (Fast Fourier Transform algorithm) was used for protein-protein interaction (Chen et al., 2003). The best ZDOCK score of protein-protein interaction and protein complex (protein+ NNK or NNAL) interaction were considered for the most suitable protein conformational pose , here protein complex (protein+ NNK or NNAL) interaction analysis will be used to explore the inhibition tendency of NNK/NNAL between two DNA repair enzymes and how these carcinogens interfering between normal protein-protein interaction as shown. For protective potential analysis AutoDock 4.0 tool was used for the docking analysis of nanoparticles (SWCNT, MWCNT and Fullerene) against NNK and NNAL.

## Results

*Molecular docking studies*

The adverse effects of NNK and NNAL on the DNA repair process enzymes were analysed after procuring all 120 PDB files of DNA repair enzymes as reported by Dhasmana et al., (2015). These enzymes of DNA repair mechanism were docked with tobacco smoke carcinogens (NNK, and its metabolites NNAL). The interaction of these enzymes with NNK and NNAL is comprehensively scrutinized in current article. NNK and NNAL have shown the binding capability with all 120 enzymes involved in DNA repair ranged from +28.15 to -7.82 Kcal/Mol and 7.81 Kcal/Mol, respectively. Docking simulations were achieved and hydrogen bonds were constructed in all the docked assemblies. The 1IRD(RCSB ID of human carbon monoxy haemoglobin) was used as a positive control (Jamal et al., 2015), to select and authenticate the most plausible biomolecular targets among the 120 DNA repair enzymes. After setting the threshold energy of positive control, the complete docking involved was screened for the selection of most probable biomolecular targets. The NNK and NNAL bind with positive control 1IRD with -6.67 and -6.30 Kcal/Mol of energy, respectively).After finishing the screening of whole docking process, the most plausible biomolecular targets of NNK and NNAL were selected. The selection was based on the interactions among 120 DNA repair enzymes, which have binding energy either equal or higher than the positive control. The NNK and NNAL binding efficiency with their probable bimolecular targets ranges from -6.49 to -7.82 Kcal/Mol and -6.79 to -7.81 Kcal/Mol, respectively. Hydrogen bond sizes and inhibition contest (Ki) were measured by using Discovery Studio Visualizer and docked .dlg file of AutoDock 4.0 respectively as mentioned in Table 1.

The current study demonstrates that NNK possesses good binding competence with top four probable biomolecular target proteins viz. 1UGH (Uracil-DNA glycosylase,-7.82Kcal/Mol), 3BTY (Alpha-ketoglutarate-dependent dioxygenase alkB homolog2, -7.50 Kcal/Mol), 3A1J (HUS1 Check Point, -7.16 Kcal/Mol) and POLG1 (DNA Polymerase Gama1, -7.07 Kcal/Mol). The NNAL metabolite has also shown good binding efficiency with top four probable bio molecular target proteins SMUG1 (– 7.81 Kcal/Mol), 1UGH (Uracil-DNA Glycosylase, -7.75 Kcal/Mol), 3BTY (Alpha-ketoglutarate-dependent dioxygenase alkB homolog2, -7.50 Kcal/Mol) and DPOLA (DNA Polymerase Alpha, -7.22 Kcal/Mol). The tobacco specific carcinogen NNK also acts as site-selective high affinity agonist for α7 nicotinic acetylcholine receptor (nAch). NNK binds with nAch by an efficiency of -5.47 Kcal/Mol energy. 

*Protein functional loss analysis by using protein-protein docking*

The probable mechanism how NNK and its metabolite NNAL may induce carcinogenesis was elucidated by implementing protein–protein docking technique. The function loss of the enzymes was evaluated by using ZDOCK scores. The ZDOCK scores of protein–protein interaction between DNA repair enzymes and their most preferential functional partners were identified (STRING database) and compared. The NNK and NNAL binding have shown to reduce the docking score of 7 important DNA repair enzymes with their respective functional partners. The maximum reduction in ZDOCK score was from 14.06 (HUS1 with RAD9) to 12.7 (NNK bound HUS1 with RAD9) followed by 14.16 (Uracil-DNA glycosylase with PCNA) to 13.64 (NNK bound Uracil-DNA glycosylase with PCNA and 16.68 (MUS81 with EME1) to 14.08 (NNAL bound MUS81 with EME1) as shown in Table 2. 

*Analysis of protective potential of nanoparticles against NNK and NNAL*

It was also explored whether nanoparticles provide any protection against NNK and NNAL. The relative orientation and binding affinities of nAch, SWCNT, MWCNT & fullerene with NNK &NNAL were compared during the current study. These elements have shown good binding properties (SWCNT with NNK -17.16 Kcal/Mol), (MWCNT with NNK -17.01 Kcal/Mol with MWCNT), (NNAL with SWCNT -15.47 Kcal/Mol), and (NNAL with MWCNT -16.25 Kcal/Mol) (Table 3) than DNA repair enzymes and nAch. The results of current study demonstrate strong likelihood of protective and preferential binding of NNK & NNAL with CNTs over with the most plausible bio molecular targets of DNA repair enzymes (1UGH with NNK -7.82 Kcal/Mol and SMUG1 with NNAL -7.81 Kcal/Mol). Fullerenes comparatively showed less binding affinities with NNK (-2.16 Kcal/Mol) and NNAL (-2.44 Kcal/Mol) (Table 4).

**Table 1:** Molecular Interaction Analysis of Probable Biomolecular Targets of NNK and NNAL among DNA Repair Enzymes Results Obtained from AutoDock 4.0

S.No	Pathway Name	Protein Name	Ligand Name	Residues	Characterized Binding Sites By LigSite Server	Binding Energy (Kcal/Mol)	Ki Value	No of H- Bond
1	Conserved DNA damage response	HUS1 [3A1J]	NNAL	Ile23,Leu26,Ala27,Cys30,Phe41,Ile42,Leu43,Cys44,Val52,Ser53,Trp55,Cys56,Tyr274	Ile23,Leu26,Ala27,Cys30,Phe41,Ile42,Leu43,Cys44,Val52,Ser53,Trp55,Cys56,Tyr274	-6.81	10.14 µM	1) B:LEU43:HN - :UNK1:O13 2) :UNK1:N14 - B:PHE41:O 3) :UNK1:H30 - B:LEU43:O 4) B:LEU43:HN - :UNK1:O13
2	Conserved DNA damage response	HUS1 [3A1J]	NNK	Ile23,Leu26,Ala27,Cys30,Phe41,Leu43,Cys44,Val52,Ser53,Trp55,Cys56,Leu272,Gln273,Tyr274	Ile23,Leu26,Ala27,Cys30,Phe41,Leu43,Cys44,Val52,Ser53,Trp55,Cys56,Leu272,Gln273,Tyr274	-7.16	5.65 µM	1) B:LEU43:HN - :UNK1:O15 2) B:TRP55:HN2 - :UNK1:O15 3) B:TRP55:HN2 - :UNK1:O7 4) :UNK1:H14 - B:LEU43:O 5) :UNK1:H15 - B:LEU43:O
3	Base excision repair (BER)	Poly(ADP-ribose) glycohydrolase (PARG) [4A0D]	NNAL	Ile726,Glu727,Gln735,Val736,Asp737,Gln754,Ile757,Arg758,Tyr792,Tyr795,Ile821 Phe902	Ile726,Glu727,Gln735,Val736,Asp737,Gln754,Ile757,Arg758,Tyr792,Tyr795	-6.96 kcal/mol	7.89 µM	1)A:ARG758:N - :UNK1:O13 2)A:ARG758:NH1 - :UNK1:N14 3):UNK1:O15 - A:VAL736:O 4):UNK1:H30 - A:GLN754:O
4	Base excision repair (BER)	NEIL3 [PM0078995]	NNK	Gly278,Tyr279,Gln280,Ser281,Lys284,His428,Ser430,Asn434,Ile438,Leu443,Arg444 Ala446,Tyr455,Gly460,Ile461,Tyr462	Gly278,Tyr279,Gln280,Ser281,Lys284,His428,Ser430,Asn434,Ile438,Leu443,Arg444 Ala446,Tyr455,Gly460,Ile461,Tyr462	-6.49 kcal/mol	17.42 µM	1):LYS284:HZ1 - :UNK1:N14 2):LYS284:HZ1 - :UNK1:O15 3):ARG444:HN - :UNK1:O7 4):TYR462:HN - :UNK1:N10 5):UNK1:H14 - :SER430:OG 6):UNK1:H15 - :GLN280:O
5	Base Excision Repair (BER)	SMUG1	NNAL	Gly83,Met84,Asn85,Pro86,Gly87,Met91 Phe98,Gly99,Glu135,Ser137,Asn163,His239	Gly83,Met84,Asn85,Pro86,Gly87,Met91,Phe98,Glu135,Ser137,Asn163,His239	-7.81 kcal/mol	1.89 µM	1):MET84:N - :UNK1:O15 2):SER137:OG - :UNK1:O13 3):UNK1:N14 - :ASN85:O :4)UNK1:O15 - :HIS239:NE2
6	Base Excision Repair (BER)	SMUG1 [PM0079127]	NNK	Met84,Asn85,Pro86,Gly87,Gly90,Met91 Val96,Pro97,Phe98,Gly99,Asn163,Asn176, His239	Met84,Asn85,Pro86,Gly87,Gly90,Met91 Val96,Pro97,Phe98,Asn163,Asn176, His239	-6.96 kcal/mol	7.88 µM	1):MET84:N - :UNK1:O7 2):PHE98:N - :UNK1:N14 3):PHE98:N - :UNK1:O15 4):ASN163:ND2 - :UNK1:N14 5):ASN163:ND2 - :UNK1:O15 6):UNK1:H15 - :ASN163:OD1
7	Base Excision repair	URACIL-DNA GLYCOSYLASE [1UGH]	NNAL	Pro120,Gly143,Gln144,Asp145,Pro146,Tyr147,Ala153,His154,Gly155,Leu156,Cys157 Phe158,Ser159,Val160	Pro120,Gly143,Gln144,Asp145,Pro146,Tyr147,Ala153,His154,Gly155,Leu156,Cys157 Phe158,Ser159	-7.75 kcal/mol	2.09 µM	1)E:TYR147:HN - :UNK1:O13 2):UNK1:O15 - E:HIS268:NE2 3):UNK1:H30 - E:ASP145:O
8	Base Excision Repair (BER)	URACIL-DNA GLYCOSYLASE [1UGH]	NNK	Pro120,Gly143,Gln144,Asp145,Pro146 Tyr147,Gln152,Ala153,His154,Gly155 Leu156,Cys157,Phe158	Pro120,Gly143,Gln144,Asp145,Pro146 Tyr147,Gln152,Ala153,His154,Gly155 Leu156,Cys157,Phe158	-7.82 kcal/mol	1.84 µM	1)E:PHE158:HN - :UNK1:O7 2):UNK1:H14 - E:SER159:O 3):UNK1:H15 – E:LEU156:O 4):UNK1:H15 - E:SER159:O
9	Homologus recombination	MUS81 [2ZIX]	NNAL	Val30Gly30,Asp307,Phe308,Ile331,Val332,Glu333,Lys335,Gln352,Lys353,Phe354,Arg355,Leu356,Lys357,Phe397	Asp307,Phe308,Ile331,Val332,Glu333,Lys335,Gln352,Lys353,Phe354,Arg355,Leu356,Lys357,Phe397	-7.06 kcal/mol	6.70 µM	1) A:ASP307:HN - :UNK1:N14 2) A:ASP307:HN - :UNK1:O15 3) A:GLN352:HE21 - :UNK1:O13 4) :UNK1:O15 - A:VAL332:O 5) :UNK1:H30 - A:GLN352:O
10	Chromatin	CHAF1 [PM0079461]	NNAL	Pro142Ser143,Ala,146,Gln734,Ile735,Leu736,Gln738,Leu739,Leu740,Leu743	Pro142Ser143,Ala,146,Gln734,Ile735,Leu736,Gln738,Leu739,Leu740,Leu743	-7.06 kcal/mol	6.69 µM	1) A:SER143:HG - :UNK1:O13 2) A:LEU736:HN - :UNK1:N14 3) A:LEU736:HN - :UNK1:O15 4) A:LEU740:HN - :UNK1:O13
S.No	Pathway Name	Protein Name	Ligand Name	Residues	Characterized Binding Sites By LigSite Server	Binding Energy (Kcal/Mol)	Ki Value	No of H- Bond
11	Direct Reversal of Damage	AlkB homolog 2 [3BTY]	NNAL	Val99,Val101,Arg110,Phe124,Ser125,Ile168,Cys169,Glu170,His171,Arg172,Asp174,Glu175,Tyr235	Val99,Val101,Arg110,Phe124,Ser125,Ile168,Cys169,Glu170,His171,Arg172,Asp174,Glu175	-7.50 kcal/mol	3.21 µM	1) :UNK1:H31 - A:ASP174:OD1
12	Direct Reversal of Damage	AlkB homolog 2 [3BTY]	NNAL	Val99,Val101,Arg110,Phe124,Ser125,Ile168,Cys169,Glu170,His171,Arg172,Asp174,Glu175,Tyr235	Val99,Val101,Arg110,Phe124,Ser125,Ile168,Cys169,Glu170,His171,Arg172,Asp174,Glu175,	-7.50 kcal/mol	3.21 µM	2) :UNK1:H31 - A:ASP174:OD1
13	Mis match repair pathway	DNA mismatch repair protein Msh3 [3THW]	NNAL	Val289,Ala290,Lys327, Ser328,Leu330 Ile331,Asn336,Glu349,Ile350,Met351,Thr352,Ser357,Ala387,Val415,Arg614	Val289,Ala290,Lys327, Ser328,Leu330 Ile331,Asn336,Glu349,Ile350,Met351,Thr352,Ser357,Ala387,Val415,Arg614	-6.95 kcal/mol	8.10 µM	1) B:ASN336:HD22 - :UNK1:O15
								2) B:THR352:HN - :UNK1:O13 3) B:ARG614:HE - :UNK1:N14 4) B:ARG614:HE - :UNK1:O15 5) B:ARG614:HH21 - :UNK1:N14 6) :UNK1:N14 - B:LEU330:O 7) :UNK1:O15 - B:LEU330:O 8) :UNK1:H30 - B:THR352:OG1
14	Mis match repair pathway	DNA mismatch repair protein Msh3 [3THW]	NNK	Arg286,Val289,Lys327,Ser328,Leu330,Ile331,Asn336,Glu349,Ile350,Met351, Thr352,Ser357,Ala387, Arg614	Arg286,Val289,Lys327,Ser328,Leu330,Ile331,Asn336,Glu349,Ile350,Met351, Thr352,Ser357,Ala387, Arg614	-6.67 kcal/mol	13.00 µM	1) B:ASN336:HD22 - :UNK1:N14 2) B:ASN336:HD22 - :UNK1:O15 3) B:ARG614:HE - :UNK1:N2 4) B:ARG614:HE - :UNK1:N14 5) B:ARG614:HH21 - :UNK1:O15 6) :UNK1:H14 - B:LEU330:O 7) :UNK1:H15 - B:LEU330:O
15	NER	GTF2H3 [PM0079474]	NNAL	Asn9,Leu10,Leu11,His48,Leu49,Ser159 Arg160,Leu190,Phe231,Pro233,Gln235 Asp236,Gln237	Asn9,Leu10,Leu11,His48,Leu49,Ser159 Arg160,Leu190,Phe231,Pro233,Gln235 Asp236,Gln237	-6.79 kcal/mol	10.52 µM	1) A:LEU49:HN - :UNK1:N9 2) A:ARG160:HH11 - :UNK1:N14 3) A:ARG160:HH11 - :UNK1:O15 4) :UNK1:N14 - A:SER159:O
16	DNA Polymerase	POLG1 [3IKM]	NNAL	Leu435,Trp441,Ile808,Gln811,Thr858,Trp859,Thr861,Ala862,Ser863,Glu873,Leu874,Lys875,Val878,Gln879,Phe1129,Leu1189	Leu435,Trp441,Ile808,Gln811,Thr858,Trp859,Thr861,Ala862,Ser863,Glu873,Leu874,Lys875,Val878,Gln879,Phe1129,Leu1189	-6.95 kcal/mol	8.01 µM	:UNK1:H30 - A:THR861:O
17	DNA Polymerase	POLG1 [3IKM]	NNK	Leu435,Trp441,Ile808,Gln811,Thr858,Trp859,Ala862,Ser863,Glu873,Leu874,Lys875,Val878,Gln879,Phe1129,Ile1131,Leu1189	Leu435,Trp441,Ile808,Gln811,Thr858,Trp859,Ala862,Ser863,Glu873,Leu874,Lys875,Val878,Gln879,Phe1129,Ile1131,Leu1189	-7.07 kcal/mol	6.61 µM	1) A:LEU874:HN - :UNK1:O7 2) A:LYS875:HN - :UNK1:O7 3) :UNK1:H14 - A:GLN879:OE1 4) :UNK1:H15 - A:GLN879:OE1
18	DNA Polymerase	DPOLA [PM0079469]	NNAL	Asn638,Tyr640,Gly641,Phe642,Glu694,Ile695,Tyr706,Asn828,Lys829,Tyr830,Lys831,Lys832,Gly833,Arg834	Asn638,Tyr640,Gly641,Phe642,Glu694,Ile695,Tyr706,Asn828,Lys829,Tyr830,Lys831,Lys832,Gly833,Arg834	-7.22 kcal/mol	5.14 µM	1) :ASN638:HD21 - :UNK1:N2 2) :ASN638:HD21 - :UNK1:N14 3) :ASN638:HD21 - :UNK1:O15 4) :ASN828:HD21 - :UNK1:N9
19	DNA Polymerase	DPOLA [PM0079469]	NNK	Asn638,Ile639,Tyr640,Gly641,Asp692,Glu694,Ile695,Asn828,Lys829,Tyr830,Lys831,Lys832,Gly833,Arg834,Lys835	Asn638,Ile639,Tyr640,Gly641,Asp692,Glu694,Ile695,Asn828,Lys829,Tyr830,Lys831,Lys832,Gly833,Arg834,Lys835	-6.90 kcal/mol	8.73 µM	1) :GLY641:HN - :UNK1:N10 2) :ASN828:HD21 - :UNK1:O15 3) :ARG834:HH12 - :UNK1:O7 4) :UNK1:H14 - :LYS832:O 5) :UNK1:H15 - :LYS829:O

**Table 2:** Normal Function loss of DNA Repair Enzymes in the Presence of NNK and NNAL Results Obtained from ZDOCK

S.No.	Pathway Name	Protein Name		ZDOCK Score	Z Rank Score
		Receptor	Ligand		
1	Nucleotide Excision Repair (NER)	GTF2H3	GTH2H4	15.24	-118.946
2	Nucleotide Excision Repair (NER)	GTF2H3-NNAL	GTH2H4	15.22	-115.817
3	MISMATCH REPAIR	3THW (MSH3)	MSH2	19.02	-109.414
4	MISMATCH REPAIR	3THW-NNAL	MSH2	18.78	-111.268
5	MISMATCH REPAIR	3THW-NNK	MSH2	18.82	-111.264
6	Conserved DNA damage response	3A1J(HUS1)	RAD9A	14.06	-103.24
7	Conserved DNA damage response	3A1J/HUS1-NNK	RAD9A	12.7	-57.149
8	Chromatin	CHAF1A2	1AXC (PCNA)	18.32	-104.796
9	Chromatin	CHAF1A2-NNAL	1AXC (PCNA)	18.26	-113.269
10	Base Excision Repair (BER)	Uracil-DNA glycosylase (1UGH)	1AXC (PCNA)	14.16	-85.938
11	Base Excision Repair (BER)	1UGH-NNK Uracil-DNA glycosylase-NNK	1AXC (PCNA)	13.64	-83.98
12	Base Excision Repair (BER)	1UGH-NNAL Uracil-DNA glycosylase-NNAL	1AXC (PCNA)	13.64	-84.738
13	DIRECT REVERSIBLE OF DAMAGE	AlkB homolog 2 (3BTY)	1AXC (PCNA)	16.14	-91.985
14	DIRECT REVERSIBLE OF DAMAGE	3BTY-NNAL	1AXC (PCNA)	16	-98.304
15	DIRECT REVERSIBLE OF DAMAGE	3BTY-NNK	1AXC	15.94	-99.769
16	Homologous recombination	MUS81(2ZIX)	EME1 (2ZIV)	16.68	-109.834
17	Homologous recombination	2ZIX-NNAL	EME1 (2ZIV)	14.08	-111.001

**Table 3:** Binding Analysis of SWCNTs, MWCNTs & Fullerene with NNK and NNAL, Results Obtained from Auto Dock 4.0

S.No.	Receptor	Ligand	Binding Energy Kcal/Mol	Ki Value
1	SWCNT	NNK	-17.16 Kcal/Mol	264.67 fM (femtomolar)
2	SWCNT	NNAL	-17.01Kcal/Mol	342.63 fM (femtomolar)
3	MWCNT	NNK	-15.56 Kcal/Mol	3.94 pM (picomolar)
4	MWCNT	NNAL	-16.25 Kcal/Mol	1.23 pM (picomolar)
5	Fullerene	NNK	-2.16 Kcal/Mol	26.19 mM (millimolar)
6	Fullerene	NNAL	-2.44Kcal/Mol	16.40 mM (millimolar)

**Table 4:** Comparative Binding Analysis of NNK and NNAL; with most Probable Biomolecular Targets which Selected among whole DNA Repair Enzymes (1UGH & SMUG1), nAch which is well known receptor for the activation of NNK induced carcinogenesis and all kind of nanoparticles discussed in this study (SWCNTs, MWCNTs& Fullerene)

S.No.	Receptor	Ligand	Energy (Kcal/Mol)
1	1UGH	NNK	-7.82
2	SMUG1	NNAL	-7.81
3	nAch	NNK	-5.47
4	Fullerene	NNK	-2.16
5	Fullerene	NNAL	-2.44
6	SWCNT	NNK	-17.16
7	SWCNT	NNAL	-15.47
8	MWCNT	NNK	-17.01
9	MWCNT	NNAL	-16.25

**Figure 5:**
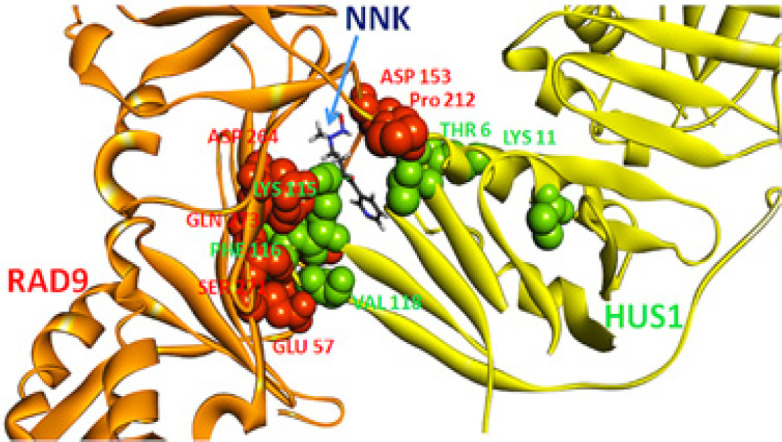
Protein–Protein Interaction of HUS1with Their Functional Partner RAD9,ZDOCK Score was Reduced from 14.06 to 12.7 and NNK Binds between the Interface ofHUS1 and RAD9. Orange and yellow coloured ribbon like structures represents the RAD9 andHUS1 proteins respectively and red and green coloured ball like structures shows binding residues of RAD9 and HUS1, which involve an intermolecular hydrogen bond. NNK is interfering between the interfaces of HUS1 andRAD9

**Figure 6:**
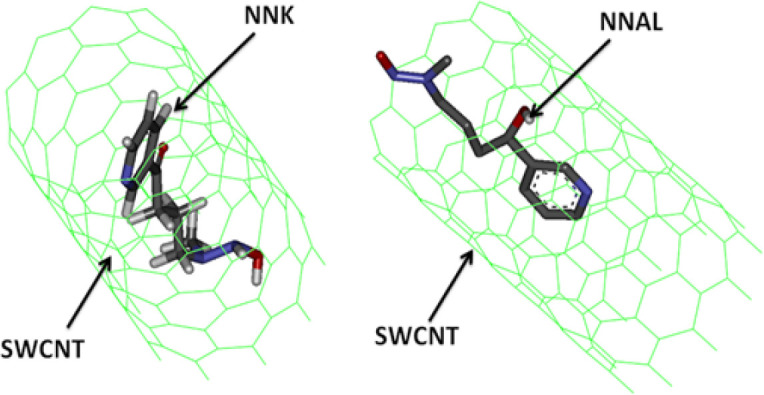
Binding Pose of NNK & NNAL with SWCNT

**Figure 7:**
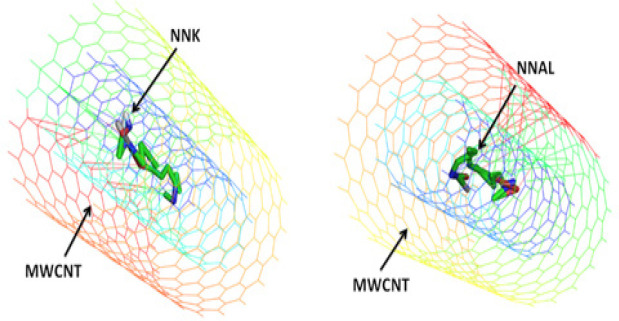
Binding Pose of NNK & NNAL with MWCNT

**Figure 8:**
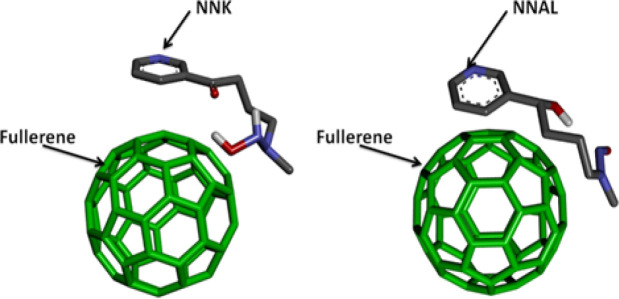
Binding Pose of NNK & NNAL with Fullerene

## Discussion

Smoking and consuming tobacco causes oral/lung cancer, is almost an established fact (Keyler et al., 2003). The consumers of tobacco are generally in contact with Tobacco Specific Nitrosamines (TSNs) like NNK and its derivative NNAL present in cigarette smoke. The NNK interacts with DNA and forms DNA adduct, accountable for the tumor development (Gharavi and Kadi, 2004). The current study attempts to explicate the process of carcinogenesis induced by cigarette smoke carcinogens NNK and NNAL by using molecular simulation techniques. The study included120 enzymes that participate in various DNA repair processes. The pdb files for all 120 enzymes were collected from the previous study conducted by Dhasmana et al.,(2015). To determine the potential biomolecular targets of TSNs, NNK and NNAL were docked with each of the 120 DNA repair enzymes. The enzymes, binding to TSNs with a score higher than the docking score of TSNs with positive control (1IRD), were considered as the potential biomolecular targets of TSNs. The assessment projected 7 potential targets of NNK, and 12 of NNAL as mentioned in the Table 1. The common biomolecular targets of both the NNK and NNAL among DNA repair enzymes were considered to be most vulnerable targets, namely, HUS1, SMUG1, Uracil-DNA glycosylase (1UGH), AlkB homolog 2 (3BTY), DNA mismatch repair protein Msh3 (3THW), POLG1 and DPOLA. 

Further, these targets were examined for the loss of molecular propertiesas a result of TSNs binding, by using an in silico ZDOCK server. The score of PPI (Protein-protein interaction) with each of the 12 probable target DNA repair proteins and their utmost standard functional partner (carefully chosen from the STRING Database) was estimated, and compared with protein complex’s (target DNA repair enzymes+TSNs) interaction with their respective potential functional partner. The results as shown by the current study clearly indicates the loss of function of 7 important DNA repair proteins (Table 2). 

The greatest decrease in ZDOCK score was found in between HUS1 and RAD9 from 14.06 to 12.7 (NNK bound HUS1 with RAD9 as shown in Figure 5). RAD9, HUS1, and RAD1 heterotrimeric complex performs dual role in checkpoint activation of cell cycle and repair of damaged DNA in human cells (Min et al., 2009), but NNK interference may acts as an obstacle for this hetromeric complex formation and can obstructs the cell cycle check point activation and hence reduce DNA repair tendency. 

The reduction in ZDOCK score was also found between Uracil-DNA glycosylase & PCNA. It was reduced from 14.16 to 13.64 (NNK bound Uracil-DNA glycosylase with PCNA). This uracil-DNA glycosylase acts as an essential DNA repair protein to eliminate uracil bases from DNA. Earlier, Kiyonari et al., (2008), reported that human nuclear Uracil-DNA glycosylase forms a complex with other proteins and starts the base excision repair by acting with proliferating cell nuclear antigen (PCNA). But, our study shows that NNK may induce interference between Uracil-DNA glycosylase and PCNA function leading to interrupt the base excision pathways. 

The normal binding of MUS81 with EME1 plays an important role in upholding genomic integrity while DNA replication occurs in recombination repair process (Taylor and McGowan, 2008). The human MUS81-EME1 complex is a flap/fork endonuclease which processes stalled replication fork intermediates (Alberto et al., 2003). This study revealed that NNAL causes the interference between MUS81 and EME1 and reduces their ZDOCK score from 16.68 to 14.08. 

Thus, the results discussed above show that TSNs may reduce the normal binding and interfere in the normal functioning of DNA repair mechanisms. This impediment in DNA repair pathway can cause damage accumulation and finally cell transformation. Therefore, the current study suggests that TSNs (NNK and NNAL) may alter the mechanisms of DNA repair pathways and enzymes execution, along with these TSNs directly damaging the DNA. These carcinogens from tobacco (like NNK) also acts as a site-selective high affinity agonist for the α7 nicotinic acetylcholine receptor or nAch (-5.47 Kcal/Mol), which controls the development of a significant small cell lung carcinoma by stimulating the secretion of serotonin. Signalling pathways/events are initiated after binding of NNK to α7 nicotinic acetylcholine receptor. The binding of NNK to its receptor, activates Raf -1 /MAP Kinase pathway, which causes phosphorylation of c-myc (Jull et al., 2001).

Following the ‘dose makes poison’ theory of Paracelsus (the father of toxicology), the low dose or concentration of substances toxic at high dose, can be used as drugs or at least as delivery vehicles. The same can hold true for CNTs (SWCNTs/MWCNTs), which at low doses, can be used for the reduction of hazardous effect of TSNs on DNA repair enzymes. The adsorption of TSNs on both the CNTs is a contribution from different adsorption sites. The strong van der waals interactions of CNTs may play an important role in scavenging NNK and NNAL from the biological system and can help to reduce the impairment of the function of DNA repair pathway enzymes (Figure 6 and Figure 7). On the other hand, fullerenes were unable to bind NNK and NNAL probably because of low surface area and atomic arrangement (Figure 8). Fullerene was also found to be poor adsorbent for analytical application in contrast to convenience adsorbents (Vergari, 2008). Contrary to fullerene, CNTs were good adsorbents. The current study also showed using in silico experiments that fullerene does not have comparatively good energy than CNTs. The shape and size of carbon nanotubes can vary in diameter, length, number of atoms and their molar weights. They are known for the adsorption of amines like tobacco specific nitrosamine (TSNs ex, NNK and NNAL). The adsorption properties of CNTs at equilibrium deprotonated by the Lewis-basic oxygen of TSNs reveals that free amine base can adsorb to SWCNTs. Hence, even at high concentrations of TSNs, there are high chances that free CNT surface would be available for the non-specific amine-CNT adsorption (Yoosefian, 2018). TSNs adsorption on nanoparticle surface could be an effect of CNTs to minimize its high free surface energy, to accomplish the atomic coordination at the surface and to establish electronic neutrality.

The possible cause for the reduced toxic effect of co-exposure may be the straight adsorption of carcinogens onto nanoparticles which renders the carcinogens unavailable to its target bio-molecules. Though, the mentioned reason of the adsorption of carcinogens to nanoparticles needs further analysis to prove the point more strongly. The scavenging potential of both the CNTs (SWCNTs and MWCNTs) can thwart the genotoxic effects of TSNs and can be applied as a chemo-preventive agent against tobacco induced carcinogenesis. And also, computer based structural analysis of biomacromolecules and CNTs can serve as a strong basis for cancer management.

In conclusion, the current analysis demonstrated the impairment of the functions of DNA repair enzymes following TSNs exposure, which can be considered as a possible cause for hampering of DNA repair mechanisms. This may result in damage build-up and finally leading to the development of cancer. The current study also proposes that carcinogens (NNK and NNAL) surely affect the process of DNA repair pathways by meddling with enzymes involved. This hazardous effect of carcinogens on DNA repair enzymes can be reduced using both CNTs. The overall adsorption on both CNTs is influenced by their adsorption sites. Due to strong van der waals interactions CNTs can scavenge the NNK & NNAL from the biological system which may further reduce the impairment of the function of DNA repair enzymes. In contrast, fullerenes were unable to bind NNK and NNAL. Their inabilities to bind NNK and NNAL may be because of low surface area. The scavenging potential of both CNTs can avert the genotoxicity posed by TSNs and therefore are proposed as chemo-preventive agent against environmental and tobacco induced carcinogenesis. This study warrants to conduct a profound analysis to describe the failure of DNA repair processes and further look into the scavenging properties of both CNTs with best suitable tools and techniques. It also suggests in vivo and in vitro experimental confirmation to validate the in-silico results acquired from the present study.
